# Supplementary material for: Overall Survival in Real-World Patients with Unresectable Hepatocellular Carcinoma Receiving Atezolizumab Plus Bevacizumab Versus Sorafenib or Lenvatinib as First-Line Therapy: Findings from the National Veterans Health Administration Database
Source: Cancers (Basel). 2024 Oct 17;16(20):3508. doi: 10.3390/cancers16203508 (PMC11506031; doi:10.3390/cancers16203508)
Supplement: Supplementary file 1 [file cancers-16-03508-s001.zip › cancers-3205416-supplementary.pdf]

Supplementary Table S1. Unadjusted median OS in the HCC treatment subgroups by liver function and etiology.

	Median overall survival (months) [95% CI]		
**Subgroup (overall n)**	**Atezolizumab + bevacizumab**	**Sorafenib**	**Lenvatinib**
**Child-Pugh class**			
A (n=1,374)	n=345<br>16.6 [12.8, 18.7]	n=681<br>10.3 [9.1,11.8]	n=348<br>11.7 [9.7,12.9]
B (n=476)	n=56<br>4.7 [3.3,6.1]	n=319<br>4.5 [3.9,5.7]	n=101<br>6.0 [3.9,7.2]
**Modified ALBI grade**			
1 (n=494)	n=138<br>22.5 [17.9, NR]	n=213<br>15.2 [12.4,17.6]	n=143<br>15.6 [12.4, 18.6]
2A (n=549)	n=140<br>15.6 [11.4, 18.5]	n=279<br>11.0 [8.4,14.0]	n=130<br>9.7 [7.1,12.8]
2B (n=655)	n=106<br>7.4 [5.1,9.3]	n=401<br>6.0 [5.1,6.8]	n=148<br>6.1 [4.4,7.3]
**Liver etiology**			
Viral (n=1229)	n=274<br>16.3 [11.4, 18.3]	n=652<br>7.8 [7.0, 8.7]	n=303<br>11.3 [8.1, 12.8]
Non-viral (n=232)	n=48<br>6.1 [4.1, 12.6]	n=130<br>8.7 [6.5, 11.5]	n=54<br>7.6 [5.3, 11.7]

Abbreviations: ALBI, Albumin-bilirubin; CI, confidence interval; HCC, hepatocellular carcinoma; NR, not reached; OS, overall survival.
